# The role of serum Dickkopf‐1 in predicting 30‐day death in severe traumatic brain injury

**DOI:** 10.1002/brb3.1589

**Published:** 2020-04-23

**Authors:** Xin Ke, Ming Yang, Jin‐Ming Luo, Yu Zhang, Xiao‐Yu Chen

**Affiliations:** ^1^ Department of Critical Medicine The Taizhou First People's Hospital Taizhou China; ^2^ Department of Neurosurgery The Taizhou First People's Hospital Taizhou China

**Keywords:** Dickkopf‐1, humans, mortality, risk factors, severity, traumatic brain injury

## Abstract

**Objective:**

Dickkopf‐1 (DKK‐1), an inhibitor of the canonical/‐catenin cascade of the Wnt pathway, was upregulated in brain tissues of hemorrhagic stroke rats, and its rising circulating levels were associated with poor prognosis of acute ischemic stroke patients. We attempted to ascertain the relationship between serum DKK‐1 levels and 30‐day death after severe traumatic brain injury (sTBI).

**Materials and methods:**

Serum DKK‐1 levels were gauged in a total of 94 sTBI patients and 94 healthy controls. Trauma severity was assessed using Glasgow Coma Scale (GCS) and Rotterdam classification based on head computerized tomography scan. Prognostic variable was 30‐day death.

**Results:**

Compared with controls, serum DKK‐1 levels were substantially elevated in patients (median value, 3.7 versus 1.0 ng/ml). Area under receiver operating characteristic curve was 0.802 (95% confidence interval (CI), 0.708–0.877) for predicting 30‐day death. Adjusted logistic regression showed that serum DKK‐1 levels above 3.7 ng/ml remained as an independent marker of 30‐day death (odds ratio, 8.573; 95% CI, 1.386–53.020) and overall survival (hazard ratio, 7.322; 95% CI, 1.320–40.622). An intimate correlation existed between DKK‐1 levels and GCS scores (*r* = −.649) in addition to Rotterdam classification (*r* = .664).

**Conclusions:**

High serum levels of DKK‐1 are closely associated with increasing severity and rising short‐term mortality of sTBI.

## Graphical abstract

Serum Dickkopf‐1 levels are significantly raised after head trauma. Serum Dickkopf‐1 levels are closely correlated with trauma severity. Serum Dickkopf‐1 levels are independently associated with 30‐day mortality.

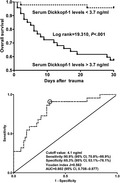

## 1 INTRODUCTION

Severe traumatic brain injury (sTBI) is a devastating type of trauma that is characterized by higher morbidity and mortality (Iaccarino, Carretta, Nicolosi, & Morselli, 2018; Jochems et al., 2018; Ostermann et al., 2018). Wnt inhibitor Dickkopf‐1 (DKK‐1), acting as a secreted protein, negatively modulates the canonical Wnt pathway (Bafico, Liu, Yaniv, Gazit, & Aaronson, 2001; Glinka et al., 1998; Niehrs, 2006). Accumulating evidence shows that elevated expression of DKK‐1 is predominantly implicated in the pathogenesis of brain diseases (Albanna & Ahmed, 2016; Caricasole et al., 2004; Dun et al., 2012; Matrisciano et al., 2011; Moors et al., 2012; Scott, Zhang, Han, Desai, & Brann, 2013). Moreover, induction of DKK‐1 was a key factor for the development of ischemic neuronal death, and blocking DKK‐1 could protect neurons against ischemic damage and decreased infarction volume in rat model with focal brain ischemia (Cappuccio et al., 2005; Mastroiacovo et al., 2009). Also, DKK‐1 levels in rat brain tissue were obviously enhanced early after intracerebral hemorrhage; inhibition of DKK‐1 could markedly ameliorated blood–brain barrier disruption and brain edema, lessened neurological deficits, increased the transcription of Wnt‐1 mRNA, and upregulated the expression of tight junction protein zonula occludens‐1 (Li et al., 2017). Clinically, serum DKK‐1 levels were substantially elevated in acute ischemic stroke, stable angina, or myocardial infarction patients (He et al., 2016; Pérez Castrillón et al., 2010; Seifert‐Held et al., 2011; Ueland et al., 2009). In addition, DKK‐1 was an independent predictor for long‐term poor prognosis of acute ischemic stroke (Zhu et al., 2019). However, there are currently no data available regarding circulating DKK‐1 levels in patients with sTBI. In our study, we intended to investigate the change of DKK‐1 in peripheral blood of sTBI patients and further determine the association between serum DKK‐1 levels and prognosis of head trauma among sTBI patients.

## 2 MATERIALS AND METHODS

### 2.1 Study participants

This study was a prospective, observational study conducted at our hospital in Taizhou, China, from August 2014 to May 2018. The inclusion criteria were to meet all of the following: (a) age ≥ 18 years, (b) sTBI (Glasgow Coma Scale (GCS) score < 9 points, not under the influence of pharmacologic agents or alcohol), (c) time from head trauma to admission ≤ 6 hr, and (d) injury severity score ≤ 9 points in noncranial aspects. The exclusion criteria were to meet one of the following: (a) surgery or trauma with recent 4 weeks, (b) previous neurological disease, such as cerebral infarction, intracerebral hemorrhage, subarachnoid hemorrhage, and brain tumors, (c) specific medication usage, such as antiplatelet and anticoagulant medication, and (d) severe diseases in other organs, such as uremia, liver cirrhosis, and malignancy. Healthy volunteers constituted controls. The study adhered to the ethical conduct of research involving human subjects by World Medical Association Declaration of Helsinki. The study was approved by the ethics committee at our hospital. Controls wrote informed consent by themselves, while patients' written informed consent was obtained from their relatives.

### 2.2 Data collection

Baseline data on demographic characteristics (age and gender), history of smoking and alcohol consumption, medical history (dyslipidemia, hypertension, and diabetes mellitus), traumatic causes (automobile/motorcycle, fall/jump, or others), and details of drug usage were collected at the time of entry into emergency department. Former smokers who had quit smoking more than 2 years ago and sporadic alcohol consumers were excluded from the smoking and alcohol analysis. Trauma severity was evaluated using the postresuscitation GCS score (Nik et al., 2018). Pupillary reactivity was observed. Upon arrival at emergency department, each patient underwent head computerized tomography (CT) scan. We recorded the following trauma‐related radiological information: abnormal cisterns, midline shift, skull‐cap fracture, skull‐base fracture, epidural hematoma, subdural hematoma, subarachnoid hemorrhage, intraventricular hemorrhage, cerebral hematoma, brain contusion, and pneumocephalus. Radiological severity was classified in light of the Rotterdam CT classification (Fujimoto, Miura, Otsuka, & Kuratsu, 2016). In addition, progressive hemorrhagic injury, acute lung injury, and post‐traumatic cerebral infarction were defined based on the previous studies (Allard et al., 2009; Bernard et al., 1994; Mirvis, Wolf, Numaguchi, Corradino, & Joslyn, 1990). All patients were followed up until death or the completion of 30 days. A 30‐day death was regarded as the study endpoint.

### 2.3 Immune analysis

Blood samples of all patients were obtained at entrance into emergency department, while those of controls were acquired at study entry. Serum C‐reactive protein levels, blood glucose, blood cell counts, and blood coagulative function were measured using the conventional methods. Acute traumatic coagulopathy was defined according to the previous reports (Franschman et al., 2012; Greuters et al., 2011). All serum samples were separated and at once frozen at −70°C until assayed. Serum DKK‐1 levels were determined in duplicate samples with a commercially available enzyme‐linked immunosorbent assay kit (R&D Systems, Inc). A standard curve was constructed from which DKK‐1 concentrations of unknown samples were quantified. Laboratory technicians who detected serum DKK‐1 were inaccessible to the clinical characteristics and outcomes of the study participants.

### 2.4 Statistical methods

Baseline characteristics of study participants were presented. Continuous variables were summarized as median (the upper–lower quartiles), and categorical variables were presented as counts (percentage). Baseline characteristics between nonsurvivors and survivors were compared using a Mann–Whitney *U* test or the chi‐squared test as appropriate. Initial univariate analysis was done to assess the statistical significance of the observed difference between nonsurvival and survival groups for each parameter. Multivariate logistic regression and Cox proportional hazards models were used to assess the association between serum DKK‐1 levels and 30‐day death when appropriate. Odds ratio (OR), hazard ratio (HR), and 95% confidence interval (CI) were calculated. All parameters that were revealed to be significant (*p* < .05) in the univariate analysis were further analyzed using multivariate analysis to identify those parameters that retained significance while accounting for all relevant variables. Differences in terms of serum DKK‐1 levels were compared among multiple groups using the Kruskal–Wallis H test. Bivariate correlations were analyzed using Spearman's rank correlation coefficient. A receiver operating characteristic curve (ROC) was constructed with serum levels of DKK‐1 as prognostic variable and 30‐day death as classification variable. Area under ROC (AUC) and its 95% CI were estimated. A combined logistic regression model was configured to assess the additive effect of serum DKK‐1 levels combined with other variables. Two‐tailed *p* < .05 was considered to be statistically significant. All analyses were performed utilizing the Statistical Package for the Social Sciences version 19.0 (SPSS Inc.).

## 3 RESULTS

During the study period, we at first assessed a total of 125 patients with sTBI based on the inclusion criteria, and further, in accordance with the exclusion criteria, we removed 31 sTBI patients because of the following reasons: (a) surgery (one cases) and trauma (two cases) with recent 4 weeks; (b) previous neurological diseases, including cerebral infarction (two cases), intracerebral hemorrhage (three cases), subarachnoid hemorrhage (two cases), brain tumors (two cases), and others (four cases); (c) specific medication usage, including antiplatelet (three cases), anticoagulant medication (two cases), and others (three cases); and (d) severe diseases in other organs, including uremia (one cases), liver cirrhosis (one cases), malignancy (two cases), and others (three cases). Eventually, 94 patients with sTBI were analyzed. Additionally, there were 94 controls composed of healthy volunteers. In terms of age and gender percentage, no significant differences existed between patients and controls.

This group of sTBI patients contained 56 males and 38 females, and their age ranged from 18 to 73 years (median, 42 years; the upper–lower quartiles, 29–53 years). Hypertension, diabetes mellitus, and dyslipidemia were found in 15, 12, and 16 patients, respectively. A total of 44 patients smoked cigarettes, and 50 patients belonged to alcohol consumers. As regards traumatic causes, most patients (45 patients, 47.9%) were traumatized by automobile/motorcycle, the second cause was fall/jump (38 patients, 40.4%), and other causes appeared in 11 patients (11.7%). Clinical severity was assessed using postresuscitation GCS scores ranging from 3 to 8, with a median value of 5 (the upper–lower quartiles, 4–7). Unreactive pupils were observed at admission among 43 patients (45.8%). All patients were admitted within 6.0 hr after trauma (range, 0.5–6.0 hr; median, 2.3 hr; the upper–lower quartiles, 2.3–3.4 hr). Abnormal cisterns occurred in 74 patients, and midline shift above 5 mm appeared in 59 patients. The other trauma‐related radiological positive appearances were listed in Table 1. There was a median value of 5 at Rotterdam CT classification (range, 3–6; the upper–lower quartiles, 4–6). In total, 58 patients underwent surgery in the first 24 hr after trauma. In addition, there were acute traumatic coagulopathy in 29 patients (30.9%), progressive hemorrhagic injury in 21 patients (22.3%), acute lung injury in 25 patients (26.6%), and post‐traumatic cerebral infarction in 11 patients (11.7%).

**Table 1:** The trauma‐related radiological positive appearances among patients with traumatic brain injury

	Count (percentage)
Skull‐cap fracture	61 (64.9%)
Skull‐base fracture	49 (52.1%)
Epidural hematoma	42 (44.7%)
Subdural hematoma	62 (66.0%)
Subarachnoid hemorrhage	66 (70.2%)
Intraventricular hemorrhage	10 (10.6%)
Intracerebral hematoma	57 (60.6%)
Brain contusion	62 (66.0%)
Pneumocephalus	30 (31.9%)

In the current study, among sTBI patients, the median value of serum DKK‐1 levels was 3.7 ng/ml, its levels ranged from 0.9 to 7.9 ng/ml, and its interquartile range was from 2.4 to 5.2 ng/ml; simultaneously, serum DKK‐1 levels of controls, with a median value of 1.0 ng/ml, ranged from 0.4 to 3.4 pg/ml (interquartile range, 0.8–1.8 pg/ml). Next, statistical analysis showed that serum DKK‐1 levels were profoundly higher in sTBI patients than in controls (*p* < .001).

In this study, there were 22 patients (23.4%) who died within 1 month following head trauma. Serum DKK‐1 levels of nonsurvivors were substantially higher than those of controls (median, 5.4 ng/ml; range, 1.8–7.9 ng/ml; interquartile range, 4.3–7.0 ng/ml versus median, 3.3 ng/ml; range, 0.9–6.9 ng/ml; interquartile range, 2.1–4.7 ng/ml). However, patients were divided into two groups in accordance with the median value of serum DKK‐1 levels (3.7 ng/ml). Just as displayed in Table 2, as compared to survivors, nonsurvivors tended to show a significantly higher proportion of serum DKK‐1 levels above 3.7 ng/ml, unreactive pupils, abnormal cisterns, midline shift > 5 mm, acute traumatic coagulopathy, acute lung injury, progressive hemorrhagic injury, and post‐traumatic cerebral infarction, as well as were likely to have markedly older age, higher serum C‐reactive protein levels, higher blood glucose levels, higher blood leukocyte count, lower admission GCS scores, and higher Rotterdam CT classification (all *p* < .05). Afterward, all the preceding variables found significant in univariate analyses were incorporated in the binary logistic regression model, and subsequently, it was revealed that serum DKK‐1 levels > 3.7 ng/ml, GCS scores, and Rotterdam CT classification retained as the three independent predictors for 30‐day death, with OR values of 8.573 (95% CI, 1.386–53.020), 0.370 (95% CI, 0.189–0.723), and 2.967 (95% CI, 1.526–5.769), respectively.

**Table 2:** Difference of clinical, radiological, and biochemical variables by 30‐day death

	Nonsurvivors	Survivors	*p* value
Gender (male/female)	12/10	44/28	.583
Age (year)	51 (35–61)	39 (28–50)	.017
Hypertension	6 (27.3%)	9 (12.5%)	.109
Diabetes mellitus	5 (22.7%)	7 (9.7%)	.144
Dyslipidemia	3 (13.6%)	13 (18.1%)	.755
Smokers	10 (45.5%)	34 (47.2%)	.884
Alcohol use	9 (40.9%)	41 (56.9%)	.187
Traumatic causes			.120
Automobile/motorcycle	11	34	
Fall/jump	6	32	
Others	5	6	
GCS scores	4 (3–4)	5 (4–7)	<.001
Unreactive pupils	21 (95.5%)	22 (30.6%)	<.001
Acute lung injury	10 (45.5%)	15 (20.8%)	.022
Post‐traumatic cerebral infarction	6 (27.3%)	5 (6.9%)	.018
Progressive hemorrhagic brain injury	9 (40.9%)	12 (16.7%)	.037
Abnormal cisterns	21 (95.5%)	53 (73.6%)	.036
Midline shift > 5 mm	18 (81.8%)	41 (56.9%)	.035
Rotterdam CT classification	6 (5–6)	4 (4–6)	<.001
Admission time after trauma (hr)	2.7 (2.3–3.4)	2.3 (2.2–3.2)	.085
Blood collection time after trauma (hr)	3.8 (3.7–4.5)	3.8 (3.2–4.9)	.534
Systolic arterial pressure (mmHg)	123 (89–134)	125 (97–144)	.321
Diastolic arterial pressure (mmHg)	80 (62–102)	75 (60–94)	.391
Acute traumatic coagulopathy	11 (50.0%)	18 (25.0%)	.026
Blood glucose levels (mmol/L)	15.3 (11.9–21.2)	12.5 (10.3–14.2)	.006
Serum CRP levels (mg/L)	19.9 (14.9–24.5)	15.5 (14.0–19.1)	.014
Blood leukocyte count (×10^9^/L)	11.9 (6.3–15.4)	9.5 (7.7–10.8)	.037
Serum Dickkopf‐1 levels > 3.7 ng/ml	20 (90.9%)	27 (37.5%)	<.001
Surgery in the first 24 hr	17 (77.3%)	41 (56.9%)	.086

Note

Data were presented as median (interquartile range) for continuous variables and as number (percentage) for categorical variables. Differences were analyzed using Wilcoxon–Mann–Whitney test, Fisher's exact test, or chi‐square test when appropriate.

Abbreviations: CRP, C‐reactive protein; CT, computerized tomography; GCS, Glasgow Coma Scale.

**Figure 1:**
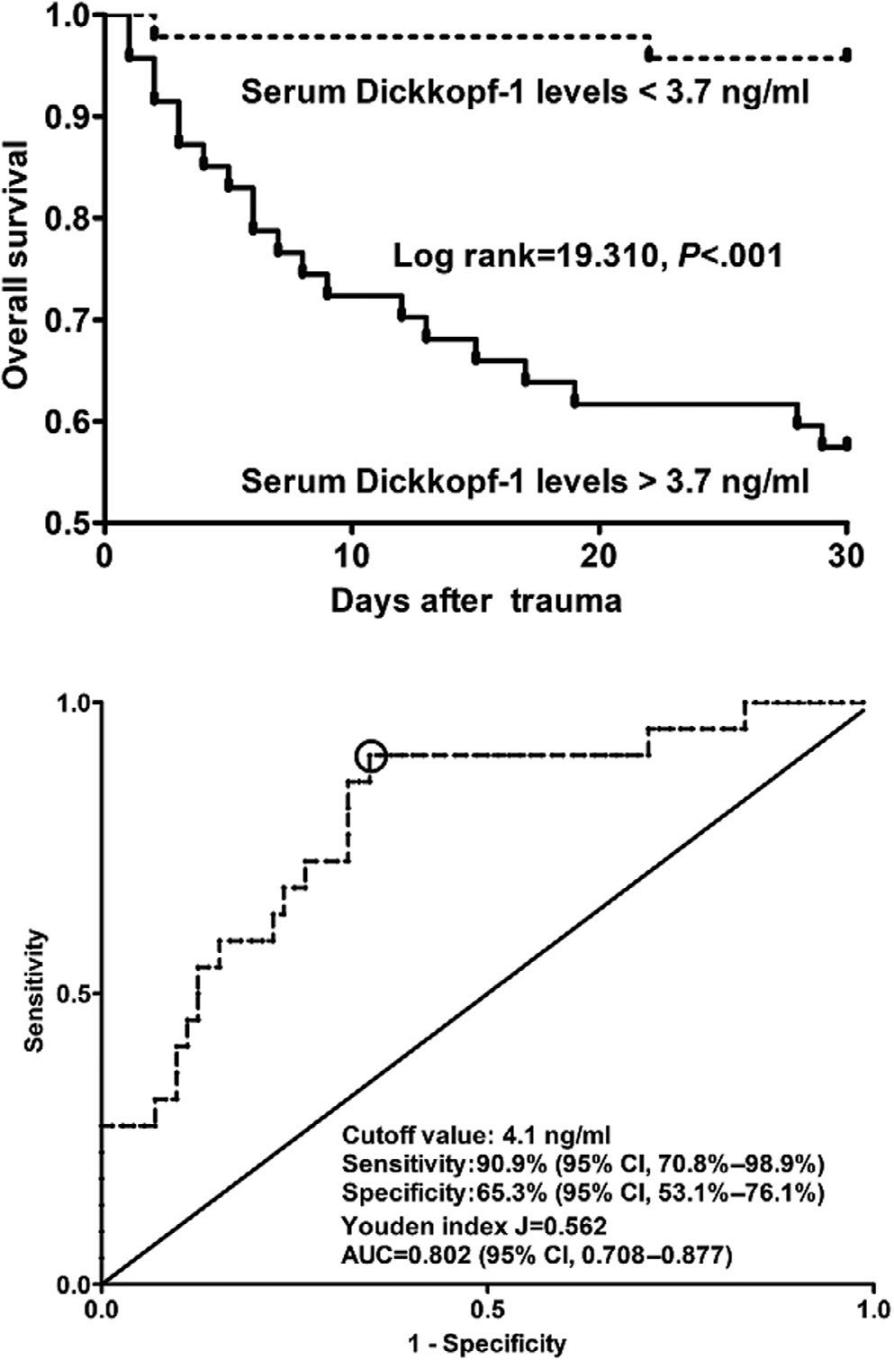
Graph showing significant difference of 30‐day overall survival time between head trauma patients with serum Dickkopf‐1 levels > 3.7 ng/ml and those who had serum Dickkopf‐1 levels < 3.7 ng/ml according to survival curve and displaying substantially high prognostic capability of serum Dickkopf‐1 levels for predicting death within 30 days after head trauma based on receiver operating characteristic curve

In this group of sTBI patients, the mean overall survival time was 25.3 days (95% CI, 23.3–27.2 days). In Figure 1, patients with serum DKK‐1 levels more than 3.7 ng/ml showed significantly shorter overall time than other remainders. Table 3 shows that the factors highly associated with pronouncedly shorter overall survival time were as following: serum DKK‐1 levels above 3.7 ng/ml, unreactive pupils, acute traumatic coagulopathy, acute lung injury, progressive hemorrhagic injury, post‐traumatic cerebral infarction, age, midline shift > 5 mm, blood leukocyte count, serum C‐reactive protein levels, blood glucose levels, admission GCS scores, and Rotterdam CT classification (all *p* < .05). Multivariate Cox's hazard proportional analysis, in which all the above‐mentioned significant variables were included, showed that serum DKK‐1 levels > 3.7 ng/ml (HR, 7.322; 95% CI, 1.320–40.622), GCS scores (HR, 0.402; 95% CI, 0.236–0.686), and Rotterdam CT classification (HR, 1.826; 95% CI, 1.027–3.246) remained independently related to 30‐day overall survival.

**Table 3:** The factors associated with 30‐overall survival after traumatic brain injury

	Hazard ratio	95% confidence interval	*p* value
Gender (male/female)	0.792	0.342–1.833	.586
Age (y)	1.037	1.008–1.067	.012
Hypertension	2.027	0.792–5.183	.140
Diabetes mellitus	2.198	0.811–5.958	.122
Dyslipidemia	0.725	0.214–2.449	.604
Smokers	0.948	0.409–2.193	.900
Alcohol use	0.562	0.240–1.315	.184
Traumatic causes			
Automobile/motorcycle	Reference		
Fall/jump	0.483	0. 167–1.392	.178
Others	0.322	0.098–1.057	.062
GCS scores	0.322	0.194–0.534	<.001
Unreactive pupils	32.556	4.372–242.421	.001
Acute lung injury	2.723	1.175–6.309	.019
Post‐traumatic cerebral infarction	3.368	1.313–8.635	.011
Progressive hemorrhagic brain injury	2.733	1.167–6.404	.021
Abnormal cisterns	6.497	0.874–48.310	.068
Midline shift > 5 mm	2.979	1.008–8.806	.048
Rotterdam CT classification	2.621	1.501–4.577	.001
Admission time after trauma (hr)	1.164	0.834–1.625	.371
Blood collection time after trauma (hr)	0.965	0.735–1.268	.800
Systolic arterial pressure (mmHg)	0.994	0.980–1.009	.430
Diastolic arterial pressure (mmHg)	1.011	0.990–1.033	.320
Acute traumatic coagulopathy	2.491	1.079–5.749	.033
Blood glucose levels (mmol/L)	1.192	1.080–1.316	<.001
Serum CRP levels (mg/L)	1.119	1.036–1.210	.004
Blood leukocyte count (×10^9^/L)	1.258	1.091–1.451	.002
Serum Dickkopf‐1 levels > 3.7 ng/ml	12.555	2.930–53.797	.001
Surgery in the first 24 hr	2.139	0.789–5.800	.135

Note

Data were yielded using the univariate Cox's proportional hazard analysis.

Abbreviations: CRP, C‐reactive protein; CT, computerized tomography; GCS, Glasgow Coma Scale.

Under ROC curve (Figure 1), serum DKK‐1 levels significantly differentiated between nonsurvivors and survivors within 30 days after trauma. Moreover, an optimal value of DKK‐1 levels (i.e., 4.1 ng/ml) was generated, which distinguished patients at risk of 30‐day death with 90.9% sensitivity and 65.3% specificity values (Youden J index = 0.562). Furthermore, its discriminatory capability was similar to those of GCS scores (AUC, 0.844; 95% CI, 0.755–0.911; *p* = .542) and Rotterdam CT classification (AUC, 0.754; 95% CI, 0.654–0.837; *p* = .349). Furthermore, DKK‐1 levels numerically improved AUC of GCS scores to 0.865 (95% CI, 0.779–0.927, *p* = .362), whereas it statistically significantly enhanced that of Rotterdam CT classification to 0.871 (95% CI, 0.786–0.931, *p* = .002).

**Figure 2:**
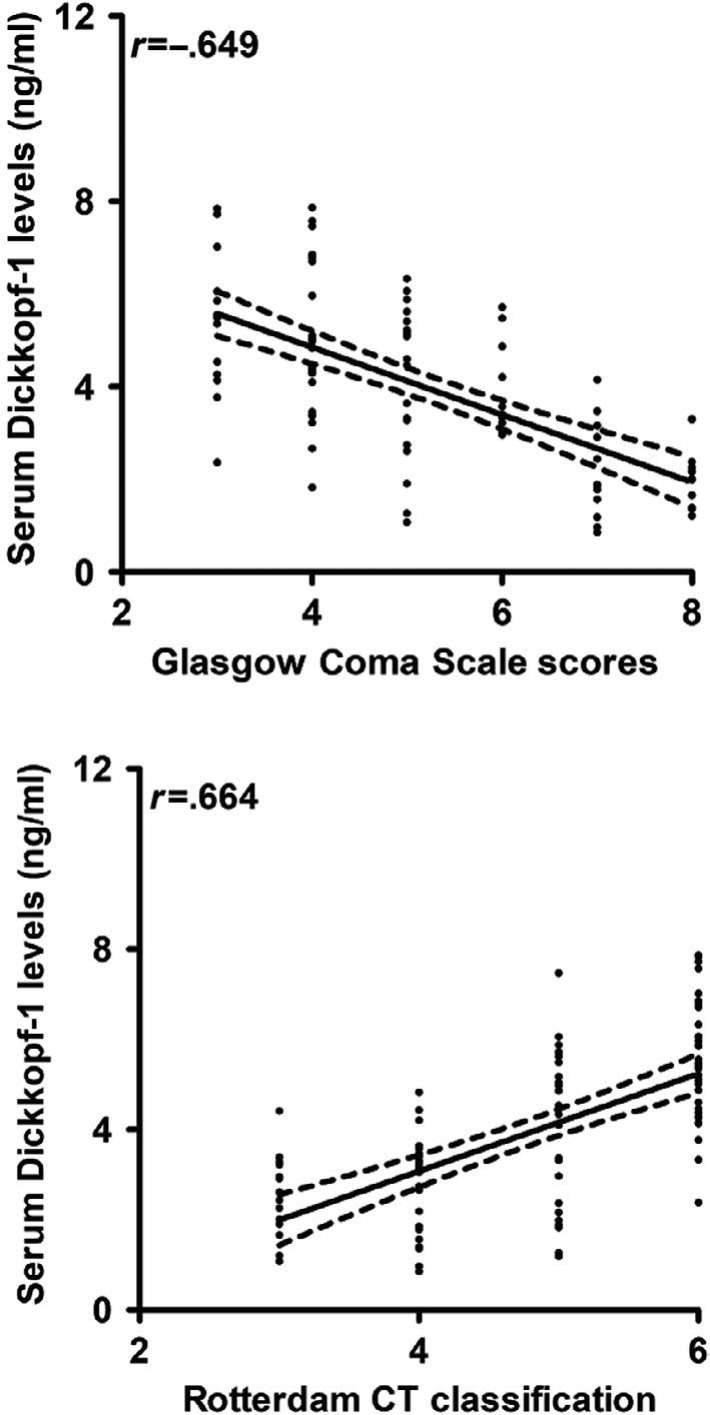
Graph depicting intimate and inverse correlation of serum Dickkopf‐1 levels with Glasgow Coma Scale (GCS) scores among traumatic brain injury patients and portraying close and positive correlation of serum Dickkopf‐1 levels with Rotterdam computerized tomography classification in patients with severe traumatic brain injury. CT means computerized tomography

When serum DKK‐1 level was designated as a categorical variable, a bivariate correlation analysis showed that serum DKK‐1 levels were closely correlated with GCS scores and CT grade (Figure 2). Similarly, while serum DKK‐1 level was identified as a continuous variable, comparative analysis among multiple groups showed that, with increasing CT grade or decreasing GCS scores, serum DKK‐1 levels were significantly raised (Table 4). Moreover, serum DKK‐1 levels were dichotomized based on its median value (3.7 ng/ml) and thereby, it was found that, patients with higher CT grade or lower GCS score had a significant higher percentage of serum DKK‐1 levels above 3.7 ng/ml (Table 4).

**Table 4:** Differences of serum Dickkopf‐1 levels by trauma severity

	Number	Serum Dickkopf‐1 levels (ng/ml)	Serum Dickkopf‐1 levels > 3.7 ng/ml
GCS scores			
3	13	5.5 (4.3–6.1)	12 (92.3%)
4	25	4.8 (4.1–6.0)	19 (76.0%)
5	19	4.6 (3.0–5.3)	11 (57.0%)
6	11	3.4 (3.1–4.6)	4 (36.4%)
7	13	1.9 (1.6–2.9)	1 (7.7%)
8	13	2.0 (1.4–2.3)	0 (0%)
*p* value		<.001	<.001
Rotterdam CT classification			
3	14	2.5 (1.9–3.2)	1 (7.1%)
4	24	3.1 (1.7–3.5)	3 (12.5%)
5	24	4.2 (2.1–5.3)	13 (54.2%)
6	32	5.3 (4.4–6.5)	30 (93.8%)
*p* value		<.001	<.001

Note

Continuous variables were reported as median (interquartile range) and categorical variables, as number (percentage). Intergroup comparisons were done by utilizing Wilcoxon–Mann–Whitney test or chi‐square test as appropriate.

Abbreviations: CT, computerized tomography; GCS, Glasgow Coma Scale.

## 4 DISCUSSION

Up to date, there have been many experimental studies detecting increased expression of DKK‐1 in brain tissues with acute brain injury (Cappuccio et al., 2005; Mastroiacovo et al., 2009). Also, it has been verified that DKK‐1 exerted a harmful effect on acute brain injury (Cappuccio et al., 2005; Mastroiacovo et al., 2009). In an intracerebral hemorrhage model, serum level of DKK‐1 did not differ between the intracerebral hemorrhage and sham groups (Li et al., 2017). Interestingly, the previous two epidemiological investigations showed that, in humans with acute ischemic stroke, DKK‐1 levels in the peripheral blood were actually higher as compared with healthy controls (He et al., 2016; Seifert‐Held et al., 2011). To the best of my knowledge, our study is the first one determining circulating DKK‐1 levels in head trauma patients. Based on our study enrolling a total of 94 sTBI and 94 controls, it was found that serum DKK‐1 levels were substantially elevated after sTBI in humans.

As regards the relationship between circulating DKK‐1 levels and disease severity of acute brain injury, only the two study has been done, in which no correlation of DKK‐1 levels was found with stroke severity (reflected by the National Institutes of Health Stroke Scale) in human acute ischemic stroke (He et al., 2016; Seifert‐Held et al., 2011). However, the patient number is little in the preceding studies (62 and 57 patients respectively) (He et al., 2016; Seifert‐Held et al., 2011). The current study included a greater sample size (namely, 94 patients). Thus, our results are more reliable and scientific. Moreover, trauma severity was assessed by the two systems including clinical and radiological scales (i.e., GCS scores and Rotterdam CT classification). In addition, in this study, serum DKK‐1 level remained as a continuous variable and was also identified as a categorical variable based on its median value. Similarly, both GCS scores and Rotterdam CT classification were transformed statistically in the same mode as serum DKK‐1 level. Whether using the bivariate correlation analysis or the nonparametric test, the close correlation of serum DKK‐1 levels with GCS scores or Rotterdam CT classification still existed. Overall, it is assumed that serum DKK‐1 levels might have the potential to assess trauma severity in patients with sTBI.

To date, there were two clinical investigations assessing the association of DKK‐1 with ischemic stroke prognosis, and no significant associations were revealed (He et al., 2016; Seifert‐Held et al., 2011), because both studies are underpowered because of small sample sizes (<100) and short follow‐up time (3 months) (He et al., 2016; Seifert‐Held et al., 2011). A recent study enrolled a large number of acute ischemic stroke patients (3,178 patients), and a poor outcome was defined as modified Rankin scale score >2 at 1 year after stroke; the data showed that circulating DKK‐1 was an independent predictor for 1‐year poor outcome (Zhu et al., 2019). In the current study, we assessed the relationship between serum DKK‐1 levels and short‐term mortality. We not only found the significantly elevated serum DKK‐1 levels in nonsurvivors within 30 days after head trauma, as compared with survivors, but also revealed the independent associations of serum DKK‐1 levels with 30‐day mortality and overall survival. Moreover, under ROC curve, serum DKK‐1 levels showed significant prognostic accuracy in discriminating nonsurvivors from survivors. In addition, serum DKK‐1 levels did not improve the prognostic power of GCS scores, while it enhanced that of Rotterdam CT classification. In summary, serum DKK‐1 might serve as a potential prognostic biomarker for short‐term mortality in human head trauma.

## 5 CONCLUSIONS

Our study provides the first evidence for a release of DKK‐1 into the circulation in patients with sTBI. We also find that elevated serum DKK‐1 levels at baseline are associated with severity and death at 30 days after sTBI, indicating that DKK‐1 may be a potential prognostic biomarker for sTBI.

## CONFLICT OF INTERESTS

The authors have no conflict of interest.

## AUTHOR CONTRIBUTION

Xin Ke and Jin‐Ming Luo designed the study and contributed to writing and editing the manuscript. Ming Yang collected clinical data and completed statistical analysis. Yu Zhang and Xiao‐Yu Chen performed follow‐up of patients, collected blood samples, and wrote and edited manuscript. All authors read and approved the final manuscript.

## Data Availability

The data that support the findings of this study are available on request from the corresponding author. The data are not publicly available due to privacy or ethical restrictions.
